# P-1114. Comparative in vitro Microbiologic Study of Two Plant-Based Wound Ointments

**DOI:** 10.1093/ofid/ofae631.1301

**Published:** 2025-01-29

**Authors:** Y Lan Truong, Joel Rosenblatt, Bahgat Z Gerges, Ying Jiang, Issam I Raad

**Affiliations:** UT MD Anderson Cancer Center, Houston, Texas; MD Anderson UT, Houston, Texas; MD Anderson UT, Houston, Texas; The University of Texas MD Anderson Cancer Center, Houston, Texas; MD Anderson UT, Houston, Texas

## Abstract

**Background:**

Plant-based wound ointments can provide antimicrobial prophylaxis without the inflammation and *cytotoxicity* associated with common antiseptic ointments that can inhibit closure of chronic wounds. *Inula Viscosa* (*IV*) is a plant with purported antimicrobial wound healing benefits. Polygalacturonic acid (PG) + caprylic acid (CAP) is a novel combination of compounds derived from plants (apple or citrus and coconut) which has been shown to be able to eradicate biofilms of bacteria and fungi. In this in vitro study, we compared the microbiologic efficacy of wound ointments containing extracts of *IV* and PG+CAP.

1% PG + 0.8% CAP vs. Inula Viscosa Wound Ointment- 48 Hour Matured Biofilm on Silicone Disks

**Figure:**
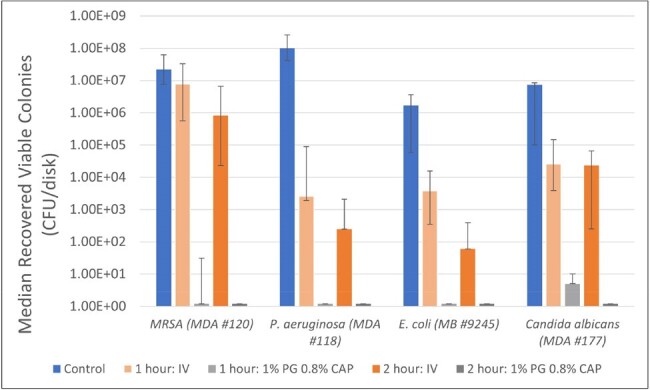

Eradication of mature biofilms of MRSA, P. aeruginosa, E. coli, and C. albicans by 1% polygalacturonic acid + 0.8% caprylic acid (PG+CAP) wound ointment and Inula Viscosa (IV) ointment after 1 hour and 2 hours of exposures. Nontreated disks were used as controls. Data is presented as the median number of recovered viable colonies from six replicates; bars indicate the range.

**Methods:**

A well-established in vitro model was used to quantify biofilm eradication. Mature biofilms of common wound pathogens isolated from cancer patients methicillin-resistant *Staphylococcus aureus* (MRSA), multidrug-resistant *Pseudomonas aeruginosa (PA)*, *Escherichia coli (EC)*, and *Candida albicans (CA)* were formed on silicone disks and then exposed to PG+CAP or commercial *IV* wound ointment (Lavior Pharma, Miami, FL) for treatment durations of one or two hours. The treated silicone disks were sonicated in neutralizing broth, serially diluted, plated and counted to enumerate viable colonies (CFU/disk). Positive controls had no wound ointment treatment.

Log10 Reduction of IV and 1% PG 0.8% CAP Wound Ointment vs Positive Control

**Figure:**
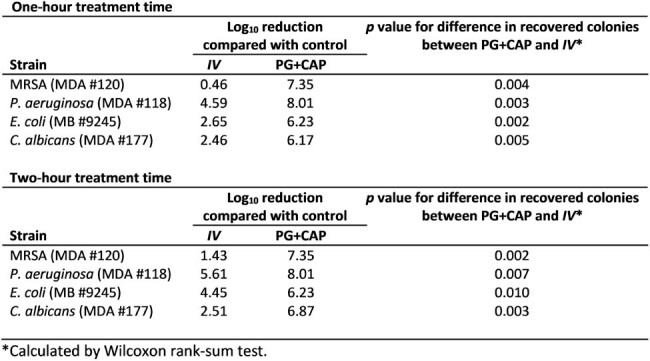

Log10 reduction of the median number of viable colonies recovered from six replicates following 1-hour and 2-hour exposure of mature biofilms of MRSA, P. aeruginosa, E. coli, and C. albicans to 1% polygalacturonic acid + 0.8% caprylic acid (PG+CAP) or Inula Viscosa (IV) wound ointment.

**Results:**

Figure 1 presents the median number of viable colonies with range bars indicating the highest and lowest number of viable colonies recovered from six replicates. Table 1 shows the median log reductions between positive control and treated samples with *p*-values comparing PG+CAP and *IV* wound ointments calculated by Wilcoxon rank sum test.

**Conclusion:**

PG+CAP fully eradicated biofilms of MRSA, *PA* and *EC* after one hour exposure. PG+CAP fully eradicated *CA* biofilm after 2-hour exposure with greater than 6-log reduction from control after 1 hour. *IV* was unable to eradicate biofilms of any of the tested pathogens after 2-hour treatment times. PG+CAP was significantly superior to *IV* (*p*< 0.05) for all pathogens at both treatment times. PG+CAP ointment merits further in vivo testing as a promising antimicrobial wound ointment.

**Disclosures:**

**All Authors**: No reported disclosures

